# Increasing paced heart rate reduces cerebral pulsatility

**DOI:** 10.1113/EP094034

**Published:** 2026-10-15

**Authors:** A. J. S. Webb, J. Thomas, O. Llwyd, N. Herring

**Affiliations:** ^1^ Department of Brain Sciences Imperial College London London UK; ^2^ Wolfson Centre for Prevention of Stroke and Dementia University of Oxford Oxford UK; ^3^ Jackie Walton Vascular Studies Unit, John Radcliffe Hospital Oxford University Hospitals NHS Trust Oxford UK; ^4^ Burdon Sanderson Cardiac Science Centre, Department of Physiology, Anatomy and Genetics University of Oxford Oxford UK

**Keywords:** hypertension, pacemaker, pulsatility, stroke

## Abstract

Cerebral pulsatility is a key index of cerebral haemodynamics, a central determinant of intravascular shear stress within intracranial vessels, and is associated with cerebral small vessel disease. It increases with a spontaneously falling heart rate, but the effect of altering heart rates on cerebral haemodynamics is unknown. We therefore tested whether increasing paced heart rate would reduce middle cerebral artery Gosling's pulsatility index (PI)on transcranial ultrasound, during emergency or elective implantation of a pacemaker for bradycardia. In 20 participants (14 males, mean age 78), paced heart rate was increased from 50 bpm or the individual's resting heart rate up to 100 bpm in 10 bpm increments. Cerebral pulsatility was strongly and inversely associated with heart rate, with an average reduction in PI of 0.07 per 10 beats per minute in unadjusted analyses, and 0.08 in mixed effects linear models adjusted for age, sex and cardiovascular risk factors (*P *< 0.001). Increasing heart rate reduced peak systolic velocity by 2.51 cm/s per 10 bpm and increased end‐diastolic velocity by 1.26 cm/s per 10 bpm, which both mediated the effect on cerebral pulsatility. However, there was no effect on mean velocity. The decrease in peak systolic middle cerebral artery velocity was linear with increasing heart rate, but end‐diastolic velocity was maximal at a heart rate of 80 bpm. Overall, cerebral pulsatility was inversely and causally associated with paced heart rate. Heart rate modification could be a potential intervention to modify cerebral pulsatility, particularly in the context of bradycardia induced by heart block.

## Abstract (highlights)

- **What is the central question of this study?**
  Do short‐term induced changes in paced heart rate have a causal effect on cerebral pulsatility, a key parameter of cerebral haemodynamics, associated with chronic cerebrovascular disease?
- **What is the main finding and its importance?**
  Increasing paced heart rate from 50 to 100 bpm reduced cerebral pulsatility due to reduced systolic cerebral blood flow velocity, and increased end‐diastolic cerebral blood flow velocity. Paced heart rate can reduce abnormally elevated cerebral pulsatility induced by bradycardia.

## 1 INTRODUCTION

Cerebral pulsatility increases with age and is tightly associated with aortic stiffness and aortic pulsatility (Webb et al., 2020, 2021) reflecting increased transmission of the aortic waveform to the brain, modified by dampening of the waveform at points of tortuosity and impedance and by the distal resistance of smaller cerebral vessels. It is highly reproducible, progresses over the age of 65 and independently predicts the risk of recurrent cerebrovascular events. However, methods to modify it independently of mean blood pressure are not clear. Cerebral pulsatility is also associated with the severity of white matter hyperintensities (Webb et al., 2012), acute lacunar infarcts, cerebral microbleeds and dilated perivascular spaces. In the ECLIPSE study a phosphodiesterase 3 inhibitor (cilostazol) (Han et al., 2013) reduced cerebral pulsatility, potentially explaining beneficial effects of cilostazol in clinical trials beyond its known antiplatelet activity (Webb, 2019). An increased pulse pressure is particularly seen in acute, severe bradycardia due to heart block, and this is therefore likely to translate into increased cerebral pulsatility.

Beat‐to‐beat cerebral pulsatility was strongly associated with reduced, concurrent heart rate, especially in older people (Webb et al., 2022). This is consistent with less reduction in diastolic flow due to a reduced length of cardiac cycle with increased heart rate, but is potentially confounded by concurrent, spontaneous changes in blood pressure. High pulsatility is therefore likely in individuals with significant bradycardia, and thus may be reduced by appropriately increasing heart rate with a pacemaker. However, the optimal heart rate to optimize cerebral haemodynamics is unclear (bhuva, 2025). However, there are very limited studies measuring the cerebral haemodynamic effects of pacemaker implantation, the effect of different pacemaker settings on cerebrovascular function, and no studies of the long‐term effects of pacemakers on vascular cognitive decline. Therefore, we tested whether increasing paced heart rate results in a reduction in cerebral pulsatility in patients undergoing acute or subacute pacemaker implantation for bradycardia.

## 2 METHODS

This study was sponsored by the University of Oxford and approved by the UK Health Research Authority and by the London Brent Research Ethics Committee (23/PR/0090). All participants provided informed consent and the research was conducted in line with the *Declaration of Helsinki*.

### 2.1 Study population

Participants were recruited from the John Radcliffe Hospital, Oxford between June 2023 and June 2024, prior to implantation of a pacemaker for symptomatic bradycardia. Eligible participants had evidence of bradycardia when symptomatic (<60 beats/min (bpm)) and a resting heart rate of <75 bpm when recruited, with at least one temporal bone window allowing transcranial Doppler ultrasound (TCD). Participants were recruited either during an acute admission for emergent pacemaker implantation for sustained bradycardia (e.g., complete heart block) or during a planned admission for intermittent symptomatic bradycardia, such as sick sinus syndrome or Mobitz Type 2 block with syncopal events or prolonged pauses on cardiac monitoring according to European Society of Cardiology guidelines (Glikson et al., 2021). People were excluded if they had recent unstable angina or myocardial infarction (within 6 months); a reduced ejection fraction (<50%); current, untreated, severe valvular heart disease; previous ventricular fibrillation or tachycardia; severe carotid stenosis (>70%); or a predisposition to intracerebral haemorrhage. Initially, the target population was 32 participants, but this was reduced to 20 participants at a pre‐planned interim analysis.

### 2.2 Experimental design

To determine the relationship between paced heart rate and cerebral pulsatility in *de novo* pacemakers, participants were tested in the cardiac catheter laboratory during pacemaker implantation, once the pacemaker was successfully implanted, usually prior to skin closure. All pacemakers were inserted under local anaesthetic without sedation. End‐tidal CO_2_ was recorded via nasal cannulae, and thus was dependent upon spontaneous ventilation.

Participants were consented at least 1 h prior to implantation and underwent a standardised interview to determine clinical and demographic characteristics. Arterial stiffness by carotid‐femoral pulse wave velocity and aortic blood pressure were determined by applanation tonometry (Syphmocor, AtCor Medical, Naperville, IL, USA). During pacemaker implantation, physiological monitoring was established including continuous middle cerebral artery blood velocity (Doppler Box, 2 MHz probe, Diamon monitoring set, DWL Compumedics, Singen, Germany), single‐lead electrocardiogram (ECG), non‐invasive continuous, beat‐to‐beat blood pressure (Finometer Nova, FMS, Amsterdam, Netherlands) and end‐tidal carbon dioxide via nasal cannulae. Insonation of the middle cerebral artery was performed on the right side via the temporal bone window, targeting a depth of 50–55 mm, distal to an identified bifurcation consistent with division of the internal carotid artery into the middle and anterior cerebral arteries. Where non‐invasive blood pressure was unreliable, repeated brachial blood pressures were recorded. Once pacing was established, most commonly ‘DDD’ pacing with an atrio‐ventricular delay of 120 ms, then participants were monitored at their spontaneous heart rate or 50 bpm. The paced heart rate was then increased by 10 bpm increments for 40–60 s, up to a maximum heart rate of 100 bpm (e.g., 50, 60, 70, 80, 90 and 100 bpm), and then in 10 bpm decrements for the same duration. This was usually performed during skin closure, or immediately afterwards.

### 2.3 Analysis

Cerebral blood velocity, heart rate and blood pressure waveforms were extracted and cleaned by a combination of automated and manual processes, as described previously (Thomas, 2023; Webb et al., 2021), to derive trough, mean and peak values for each heart beat. Briefly, ectopic heart beats were automatically detected by excessively short inter‐beat intervals (≤3 SD), with linear interpolation of blood pressure and blood flow velocities for beats preceding and following the ectopic. Waveforms were visually reviewed, with linear interpolation of errors, whilst marked outliers were excluded from further analysis. The primary outcome was middle cerebral artery (MCA) pulsatility index (MCA‐PI, Gosling's pulsatility index). Secondary outcome measures included diastolic, mean and systolic blood pressure (DBP, MBP and SBP); and end‐diastolic, mean and peak systolic MCA velocities (EDV, MV and PSV).

The primary, prespecified analysis was determined as the relationship between heart rate and MCA‐PI, and with each secondary outcome, by a linear regression across all beats for each individual, then averaged between individuals. For a secondary, prespecified confirmatory analysis, mean heart rate, pulsatility and cerebral blood velocity were determined for the best 10 beats during each fixed heart rate during the heart rate escalation/de‐escalation paradigm. These beats were determined by visual inspection in each monitoring period as the latest consecutive 10 beats in the monitoring period including no artefacts in the TCD and blood pressure waveforms. Where two periods of monitoring were available at each heart rate, the resulting cerebral blood flow velocity (CBFV) and blood pressure (BP) values were averaged across the two periods. Associations between mean values during each monitoring period were again determined for each participant by linear regression. Thirdly, the population average during each heart rate was determined for each heart rate period, both as the average absolute value and as the proportional change to the same value for each individual at 80 bpm.

The final prespecified confirmatory analysis was carried out as mixed effect linear models with an autoregressive, autocorrelation design to allow for repeated measures within each individual, with individual participants as random effects, and heart rate as fixed effects. This was performed for both approaches to modelling the association between heart rate and each outcome, with the primary analysis using all beats individually, and secondarily using the best 10 beats in each monitoring period. This was performed to determine the contribution of heart rate to each outcome measure across the whole population, unadjusted and adjusted for clinical features (age, sex, aortic stiffness, hypertension, diabetes, smoking, aortic stiffness, etc.) to adjust for potential confounding of the relationship inducing differences between heart rate and cerebral pulsatility, particularly with age. Finally, mediation analyses were performed for the mixed‐effects models as a *post hoc* analysis at an individual and group level to determine whether changes in systolic or diastolic cerebral blood flow velocity had a dominant effect in mediating the change in cerebral blood velocities and cerebral pulsatility, and whether this pattern was consistent for changes in blood pressure. These mediation analyses were performed for mixed effects models with individual heart beats as fixed effects, or grouped by the estimated average during the best 10 beats during each monitoring period. All mediation analyses for competing mediators (PSV and EDV or SBP and DBP) were performed within a Structural Equation Modelling framework to define each pathway of effect (direct or via each of the two possible mediators) with the R lavaan package.

All analyses were performed combining heart rates from the period of increasing heart rate and decreasing heart rate ramp, both for individual beats and when averaged across the best 10 beats (providing two estimates for each heart rate period in each linear regression or mixed effects model).

All analyses were performed in MATLAB R2024a (MathWorks, Natick, MA, USA) using in‐house software, or in RStudio with the packages tidyverse, psych, rms, nlme, lme4, readxl, lavaan.

## 3 RESULTS

Twenty participants were recruited (14 male, 6 female), of whom eight underwent repeated testing after at least 8 weeks. Participants were slightly older than the population median age of pacemaker implantation, with 45% recruited during an acute admission (Table 1). A history of hypertension or use of hypertensives was present in the majority of participants, and the mean blood pressure at ascertainment was elevated.

**TABLE 1:** Population demographics.

Variables	Baseline only	Follow‐up testing study	*P*
*n*	20	8	
Age	78 (7.1)	79 (7.2)	0.526
Male	14 (70)	6 (75)	1.000
Acute admission	9 (45)	2 (25)	0.313
CHB	10 (50)	4 (50)	1.000
Mobitz Type 2	5 (25)	2 (25)	1.000
Sinus node disease	1 (5)	1 (12.5)	0.492
Slow AF	2 (10)	0 (0)	0.361
Junctional bradycardia	2 (10)	2 (25)	0.313
Diabetes	1 (5)	1 (12)	0.834
Hypertension	17 (85)	7 (87)	1.000
Smoker	8 (40)	8 (100)	0.461
White	20 (100)	8 (100)	—
Alcohol (units)	2.5 (0–10)	9 (0–16.5)	0.098
Stroke	0 (0)	0 (0)	—
Previous brain imaging	7 (35)	4 (50)	0.503
Antihypertensives	17 (85)	7 (88)	0.701
Systolic BP (mmHg)	155 (21)	157 (26)	0.712
Diastolic BP (mmHg)	73 (12)	75 (12)	0.496
Baseline heart rate (bpm)	54 (13)	60 (12)	0.129
PWV (m/s)	10 (5.1)	11.3 (6.6)	0.329
End‐tidal CO_2_ (%)	4.06 (0.35)	3.75 (0.64)	0.150
Mean flow velocity (cm/s)	43.8 (9.1)	43.8 (9.6)	0.997
Pulsatility Index	0.85 (0.14)	0.85 (0.14)	0.991

*Note*: Proportions are given as *n* (%), continuous variables as mean (SD) or median (IQR).

Abbreviations: AF, atrial fibrillation; CHB, complete heart block; PWV, pulse wave velocity.

Mean cerebral blood velocities were consistent for a population of this age (Webb et al., 2020) with a modestly elevated MCA‐PI across the population (Table 2), with similar values at follow‐up. There were no significant differences between participants admitted acutely or electively, although there was a slightly higher MCA‐PI and higher blood pressure in the acute group (Table 2).

**TABLE 2:** Average values of cerebral blood velocity indices and blood pressure at 80 bpm for the whole population and stratified by acute or elective admission.

	All		Acute		Elective		
Variable	Mean	SD	Mean	SD	Mean	SD	*P*
*n*	17	17	9	9	8	8	
PSV (cm/s)	68.38	14.21	72.40	13.75	65.26	14.55	0.333
EDV (cm/s)	31.50	7.10	31.40	6.14	31.57	8.14	0.961
MFV (cm/s)	43.79	9.08	45.07	8.25	42.80	10.05	0.629
PI	0.85	0.14	0.92	0.12	0.80	0.13	0.072
SBP (mmHg)	154.00	23.46	171.83	25.26	145.08	18.42	0.198
DBP (mmHg)	80.94	9.83	85.50	1.80	78.67	11.60	0.213
MBP (mmHg)	105.30	13.40	114.28	9.60	100.81	13.34	0.137
PP (mmHg)	73.06	17.53	86.33	23.50	66.42	10.59	0.276

Abbreviations: DBP, diastolic blood pressure; EDV, end diastolic velocity; MBP, mean blood pressure; MFV, mean flow velocity; PI, pulsatility index; PP, pulse pressure; PSV, peak systolic velocity; SBP, systolic blood pressure.

Paced heart rate was strongly associated with MCA‐PI, with an inverse association across average values at each specified paced heart rate (Figure 1), and very similar relationships when averaging β‐coefficients from linear regressions in each individual, with an average 0.07 reduction in MCA‐PI per 10 bpm (Table 3). There was a striking consistency between individuals when standardising values relative to 80 bpm (Figure A1). Effects were consistent in a mixed effect linear model resulting in a 0.08 reduction in PI for every 10 bpm (Table 3, Figure A2), including after adjustment for age, sex and cardiovascular risk factors, with similar effects when including all individual heart beats rather than averaged values at each heart rate level (Table A1). These effects were similarly present in the participants’ studies at the follow‐up visit (Figure A3).

**TABLE 3:** Associations between heart rate and blood pressure or cerebral blood velocity parameters.

	β (linear)								
Outcome	Mean	SD	*P*	Unadjusted β (LME)	*P* (LME)	Age/sex adjusted β (LME)	*P*	Full adjusted β (LME)	*P*
PSV (cm/s)	−3.18	3.00	<0.0001	−2.56	<0.0001	−2.54	<0.0001	−2.51	<0.0001
EDV (cm/s)	0.61	1.23	0.980	1.24	<0.0001	1.25	<0.0001	1.26	<0.0001
MFV (cm/s)	−0.65	1.42	0.030	−0.06	0.828	−0.05	0.851	−0.04	0.894
PI	−0.07	0.04	<0.0001	−0.08	<0.0001	−0.08	<0.0001	−0.08	0.000
SBP (mmHg)	−8.40	11.54	0.004	−1.27	0.555	−1.20	0.578	−0.58	0.790
DBP (mmHg)	1.46	6.53	0.790	3.24	0.003	3.21	0.003	3.27	0.003
MBP (mmHg)	−1.83	7.07	0.176	1.42	0.232	1.39	0.245	1.77	0.152
PP (mmHg)	−9.86	10.13	<0.0001	−4.93	0.013	−4.88	0.014	−4.33	0.030

*Note*: Results are presented as the average β‐coefficient from linear regressions for each of the 20 participants and its standard deviation (SD), and as the β‐coefficient from linear mixed models including all individuals, unadjusted and adjusted for age and sex, or age, sex and cardiovascular risk factors. Results are derived from the average values for each individual at each defined heart rate. Abbreviations: DBP, diastolic blood pressure; EDV, end diastolic velocity; LME, linear mixed effect; MBP, mean blood pressure; MFV, mean flow velocity; PI, pulsatility index; PP, pulse pressure; PSV, peak systolic velocity; SBP, systolic blood pressure.

The effect of heart rate on MCA‐PI reflected a significant decrease in PSV in individual tests and on average (Table 3). Although there was not a significant effect on EDV when averaging individual relationships, due to inter‐individual variability (Figure 1), there was a significant increase in EDV when analysed with a mixed effect linear model, including after adjustment. However, this relationship was less linear, with an increase in EDV with HR below 80 bpm, but no increase, or a decrease, above this heart rate. Overall, there was a very small reduction in mean cerebral blood velocity of less than 1 cm/s per 10 bpm.

The effect of heart rate on cerebral blood velocity parameters matched the effect on blood pressure indices, with a marked reduction in pulse pressure in particular (Figure A4, Table 3), reflecting a marked reduction in systolic blood pressure (8 mmHg per 10 bpm) and increase in diastolic blood pressure, including when averaging individual results or when estimated by mixed effect models. There was an increase in diastolic blood pressure in mixed effect linear models, but there was no overall significant effect on the mean blood pressure with either analysis.

The relationships between heart rate and either cerebral pulsatility, or cerebral blood velocity or blood pressure parameters were consistent across demographic groups and cardiovascular risk factors (Table 4), although the study is underpowered to assess this. There was a weak trend to a more negative relationship between heart rate and cerebral pulsatility at older ages, and a significantly greater increase in EDV with increasing heart rate at older ages. A higher baseline absolute cerebral blood velocity also predicted a more negative relationship between heart rate and PSV and less of an increase in EDV but was not associated with a difference in the relationship with cerebral pulsatility (Table 4). There was no association between baseline clinical characteristics and blood pressure indices (Table A2), and the effect of age or absolute cerebral blood velocity on the association between heart rate and PSV or EDV was unaffected by adjustment for age, sex or baseline blood pressure (Table A3).

**TABLE 4:** Associations between demographic variables or mean cerebral haemodynamic variables at baseline with the individual participant level associations (β‐coefficients) between heart rate and cerebral blood velocity parameters from linear regressions.

	HR–PSV	*P*	HR–EDV	*P*	HR–MV	*P*	HR–PI	*P*
Age	−0.050	0.634	0.097	0.014	0.048	0.337	−0.002	0.080
Male	−0.163	0.919	0.134	0.838	0.035	0.963	0.001	0.981
Diabetes	−1.032	0.750	−0.922	0.484	−0.959	0.531	−0.021	0.621
Hypertension	−0.817	0.730	−1.336	0.154	−1.163	0.292	0.018	0.565
Smoker	−0.143	0.967	2.671	0.056	1.733	0.288	−0.063	0.157
Alcohol	−0.002	0.985	−0.028	0.410	−0.019	0.629	0.000	0.788
PWV (m/s)	−0.234	0.168	0.086	0.203	−0.021	0.776	−0.004	0.159
Acute admission	0.608	0.703	0.209	0.748	0.342	0.651	0.003	0.883
Systolic BP (mmHg)	−0.055	0.250	−0.026	0.174	−0.036	0.111	0.000	0.810
Diastolic BP (mmHg)	−0.135	0.221	−0.018	0.718	−0.057	0.310	−0.002	0.398
PSV (cm/s)	−0.134	0.003	−0.017	0.454	−0.056	0.036	0.000	0.614
EDV (cm/s)	−0.266	0.006	−0.013	0.776	−0.098	0.086	−0.002	0.243
MFV (cm/s)	−0.217	0.003	−0.019	0.588	−0.085	0.045	−0.001	0.391
Pulsatility index	1.103	0.846	−1.216	0.613	−0.443	0.886	0.092	0.194

*Note*: Reported values represent a β‐coefficient from an unadjusted linear regression between each predictor and the β‐coefficients for each individual for the relationship described in each column. Abbreviations: BP, blood pressure; EDV, end diastolic velocity; MFV, mean flow velocity; PSV, peak systolic velocity; PWV, pulse wave velocity.

As expected, the effect of heart rate on cerebral pulsatility was almost entirely mediated by the effect of heart rate on peak systolic and end‐diastolic velocity, as PI is calculated from these two indices (total direct effect HR to PI = −0.01 per 10 bpm; total indirect effect = −0.07 per 10 bpm). However, there was a slightly greater effect via systolic than diastolic blood pressure when modelled together (HR–SBP–PI = −0.04; HR–DBP–PI = −0.03), with very similar results when using all heart beats individually or using the average values during each specified heart rate. Effects of heart rate on blood pressure indices mediated some of the effect on cerebral pulsatility (indirect effect = −0.04 per 10 bpm, *P* = 0.002; direct = −0.03, *P* = 0.004), with more effect of DBP than SBP (HR–DBP–PI = −0.03, *P* = 0.03; HR–SBP–PI = −0.02, *P* = 0.15).

The effects of heart rate on systolic versus diastolic cerebral blood velocity, and thus cerebral pulsatility, are also evident with assessment of the average pulse waveform in the brain at each specified heart rate (Figure 2). With a sequential increase in heart rate, there is a systematic reduction in the systolic peak across the range of heart rates tested, and particularly with a reduction in the augmentation of the pressure waveform and the anacrotic notch. In contrast, end‐diastolic velocity increases between 50 and 80 bpm, but above this level, there is no further increase in EDV, and a potential fall as heart rate increases above 80 bpm (Figures 1 and 2).

**FIGURE 1:**
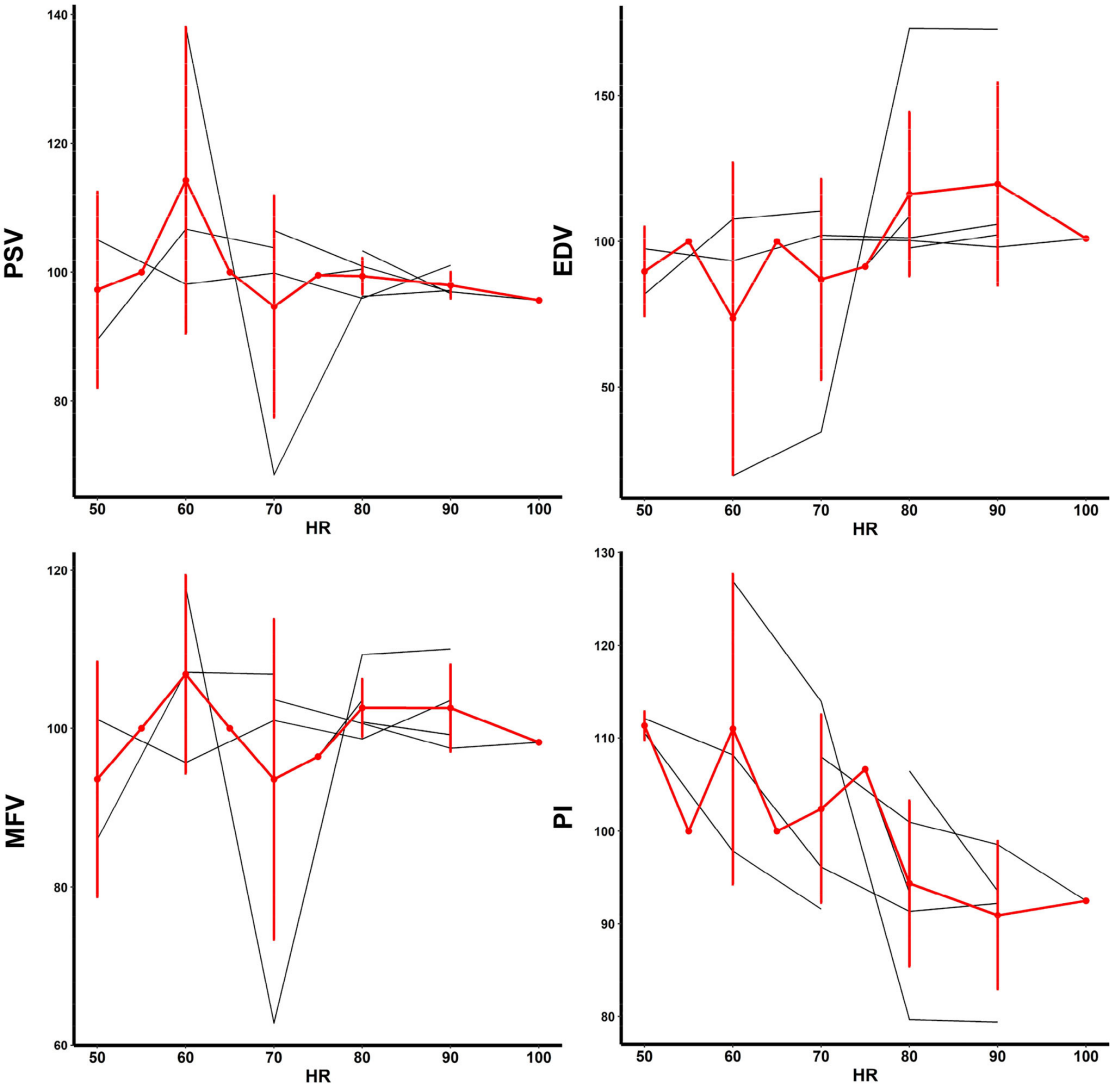
Unstandardised relationship between paced heart rate and haemodynamic parameters on TCD. Results are shown for each individual participant and the average across participants at each defined heart rate (red), as a percentage of the value at 80 bpm, for peak systolic velocity (a), end‐diastolic velocity (b), mean flow velocity (c) and pulsatility index (d).

**FIGURE 2:**
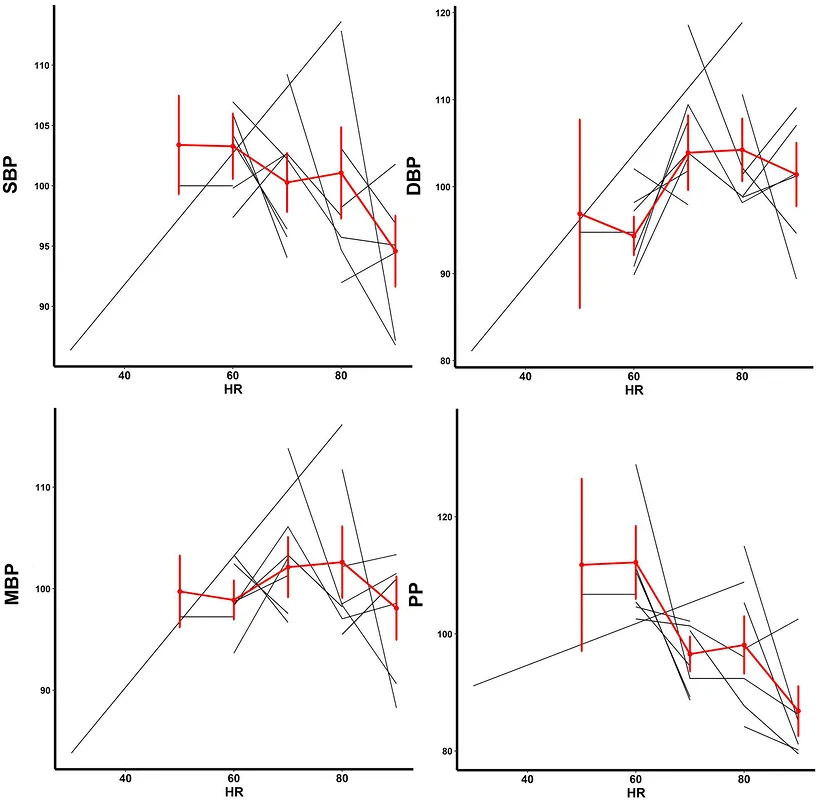
Average middle cerebral arterial blood flow velocity waveform at each defined heart rate. Results are standardised as the difference from the individual mean value at 80 bpm, expressed as a percentage of the pulsatility index at 80 bpm.

## 4 DISCUSSION

In a cohort of 20 patients requiring implantation of a pacemaker, there was a strong association between increased paced heart rate and a significant reduction in cerebral pulsatility, with an average 0.08 reduction in PI for every 10 bpm increase in heart rate. This reflected a systematic reduction in peak systolic blood velocities due to reduced augmentation, and a rise in end‐diastolic flow up to 80 bpm. However, there was very limited effect on mean cerebral blood velocity. There were consistent effects on blood pressure, with a reduction in pulse pressure and systolic pressure and an increase in diastolic pressure up to 80 bpm but limited effect upon mean blood pressures. Although effects of heart rate on cerebral blood flow are dependent on effects on aortic blood flow, the measured effects on peripheral blood pressure only partially mediated the relationship between heart rate and cerebral blood velocity.

The relationship between heart rate and cerebral pulsatility is driven by both a reduction in systolic blood flow and an increase in diastolic flow, with effects of heart rate affecting aortic blood flow which is transmitted to the brain as the principal determinant of cerebral blood flow (Webb et al., 2012). As heart rate rises, the R–R interval is reduced and the period for diastolic pressure to fall is reduced. This is enhanced in older patients with increasing aortic stiffness (Webb et al., 2012) due both to more rapid transmission of the reverse travelling wave from the periphery, and a diminished Windkessel effect in the aorta as the elasticity of the aorta is reduced. However, the mediation of the relationship between heart rate and pulsatility by systolic and diastolic blood flow velocities confirms a slightly greater effect of systolic blood flow compared to diastolic. Although pulsatility is inevitably mediated by PSV and EDV as it is calculated through these indices, this analysis confirms that the influence of heart rate is not only mediated by this mechanism, but also by secondary effects on systolic blood flow.

The limited mediation of the relationship between blood pressure and cerebral pulsatility may reflect the greater effect of heart rate on the aortic blood flow and cerebral circulation rather than on peripheral blood pressure. At the same time, there is reduced time for atrial filling and a reduced atrial ‘kick’, thus limiting the maximum systolic output and the apparent augmentation of the cardiac waveform. Once heart rate rises above 80 bpm, diastolic flow no longer rises but starts to reduce in parallel to the systolic flow, likely due to reduced filling time and an overall reduction in stroke volume.

Cerebral pulsatility is a not only a key index of cerebrovascular function, but may also have direct detrimental effects on cerebrovascular disease, and thus could reflect an increased cerebrovascular risk in individuals requiring a pacemaker due to heart block induced bradycardia. A previous study demonstrated that concurrent, beat‐to‐beat cerebral pulsatility was inversely associated with concurrent heart rate, with a stronger effect in older patients with established small vessel disease on brain imaging (Webb et al., 2022). Cerebral pulsatility was increased by treatment with a rate‐reducing beta‐blocker in 10 healthy volunteers compared to amlodipine (Webb & Rothwell, 2016) whilst cilostazol, which increases heart rate by PDE3 inhibition in the heart, modestly reduced cerebral pulsatility by 0.04 in the ECLIPSE trial of patients with a lacunar stroke (Han et al., 2013). The potential for cerebrovascular harm in block‐induced bradycardia could therefore reflect an increased risk of cerebrovascular injury and cognitive decline. Cerebral small vessel disease is the most prevalent cause of vascular cognitive impairment (Simoni et al., 2012) and is strongly associated with both cerebral pulsatility and a low end‐diastolic blood flow (Shi et al., 2020) as well as hypoperfusion of the brain in the white matter (Markus et al., 2000). However, the direction of causality is unclear without an intervention that specifically reduces cerebral pulsatility. Thus, the significant 0.08 reduction in MCA‐PI by increasing paced heart rate by 10 bpm and the reduction of people with a PI above 1, with a heart rate below 50, has potential both to test the hypothesis that pulsatility is a causative driver of cerebral small vessel disease and to test whether increased heart rate has potential as an intervention to reduce the risk of cognitive decline in patients with pacemakers.

Of the 30,000 pacemakers implanted annually in the UK (bhuva, 2025) and almost 1 million globally, the majority are implanted in people over the age of 70 for bradycardic indications such as sick sinus syndrome and atrio‐ventricular conduction system disease, as well as heart failure. These patients are therefore at an increased risk of cerebral hypoperfusion due to either intermittent bradycardia or ventricular systolic dysfunction reducing cardiac output, and thus may be at an increased risk of cerebral small vessel disease and vascular cognitive decline (Ovsenik et al., 2021). Conventionally pacemakers are programmed to a lower base rate to minimize pacing where possible and maintain battery life, although this may not be an issue in octogenarians with less than 10–15 years’ life expectancy given the battery duration of most modern pacemaker devices. Moreover, whilst a lower resting heart rate in younger patients is associated with cardiovascular fitness and lower mortality (Cole et al., 1999; Jouven et al., 2005), this is unlikely to apply to octogenarians with isolated cardiac conduction disease but no angina and otherwise normal left ventricular function. Therefore, increasing paced heart rate to more physiological levels in patients at risk of cerebrovascular injury, but with normal cardiac function, has the potential to significantly reduce the clinical burden of the disease in this high‐risk group, warranting further investigation.

This study benefitted from careful physiological testing, in a controlled environment and a highly consistent relationship between the primary exposure and the primary outcome, but there were limitations to the study. Firstly, this was a small cohort, although there was a very consistent relationship between heart rate, cerebral pulsatility and absolute cerebral blood velocity. However, the small size of the cohort means that the study is underpowered to determine whether clinical and demographic risk factors interact with these relationships, and in which groups the relationship between heart rate and cerebral pulsatility may be particularly important. Secondly, the cohort were only studied immediately after pacemaker implantation, with increased pacing rates for less than 10 min. Although this provides an advantage as it tests the effect of increased heart rate in a heart not previously paced, it is unknown whether there is physiological adaptation that could counteract the effect of increased heart rate during sustained pacing. This needs testing in further studies. Thirdly, continuous measurement of blood pressure during acute pacemaker implantation in the catheter laboratory was limited by peripheral vasoconstriction affecting reliability of measurement from the finger, limiting analysis of the effect of heart rate on the blood pressure waveform and the mediating effect of blood pressure on cerebral blood flow. Fourthly, brain imaging was not available in sufficient participants to draw any conclusions about the relationship between heart rate and cerebral pulsatility in people with established cerebrovascular disease, as suggested in previous studies.

Overall, this experimental medicine study identifies a novel potential mechanism by which a key index of cerebral blood flow is controlled. The strength of the physiological relationship is highly robust despite the small sample size, but there is a need for more detailed studies of the effect of longer duration pacing at a higher heart rate to determine whether the effect of higher paced heart rates is sustained, and whether it is associated with any detrimental effects, such as increased cardiac strain. However, if pacing at a higher rate is safe, tolerable and sustained, it provides a simple intervention that could be tested in a large population of patients. This could be tested either in people with a new pacemaker or a pacemaker already in situ, including in older patients at an increased risk of cerebrovascular disease. However, such an intervention may benefit from further analysis of the optimal paced rate, and how this may vary by patient characteristics, time of day and activity levels.

### 4.1 Conclusion

Cerebral pulsatility was reduced by increasing paced heart rate at the time of pacemaker implantation. This reflected a reduction in systolic cerebral blood velocity and an increase in diastolic cerebral blood velocity, up to 80 bpm with similar but partially independent effects on blood pressure. This presents a novel approach to modify cerebral pulsatility that needs testing in systematic clinical trials.

## AUTHOR CONTRIBUTIONS

All authors contributed to the acquisition, processing and analysis of data, and contributed to the writing and editing of the final manuscript. A.J.S.W. and N.H. conceived of the study, whilst A.J.S.W. was responsible for primary analysis and initial drafting of the manuscript. All authors have read and approved the final version of this manuscript and agree to be accountable for all aspects of the work in ensuring that questions related to the accuracy or integrity of any part of the work are appropriately investigated and resolved. All persons designated as authors qualify for authorship, and all those who qualify for authorship are listed.

## CONFLICT OF INTEREST

None declared.

## GENERATIVE AI STATEMENT

No AI tools have been used in the analysis, writing or images in this article.

## Figures and Tables

**FIGURE A1:**
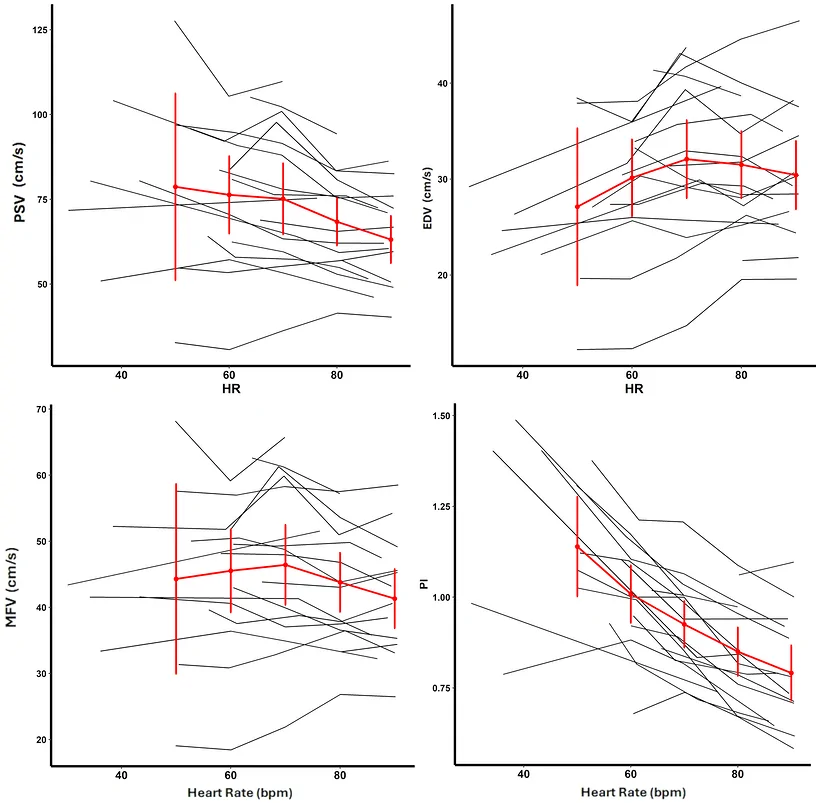
Standardised relationship between paced heart rate and haemodynamic parameters on TCD. Results are shown for each individual participant and the average across participants at each defined heart rate (red) for peak systolic velocity (a), end‐diastolic velocity (b), mean flow velocity (c), and pulsatiliy index (d).

**FIGURE A2:**
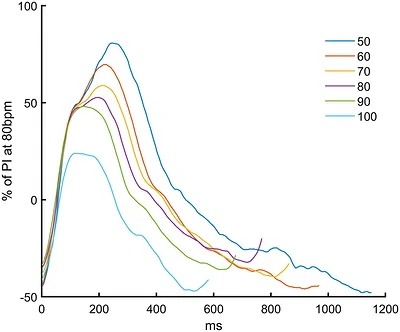
Modelled relationship between paced heart rate and haemodynamic parameters on TCD. Results are shown for the linear regression for individual participant and the average linear association from an adjusted linear mixed effect model across all participants, derived from individual heart beats for each individual, for peak systolic velocity (a), end‐diastolic velocity (b), mean flow velocity (c), and pulsatiliy index (d).

**FIGURE A3:**
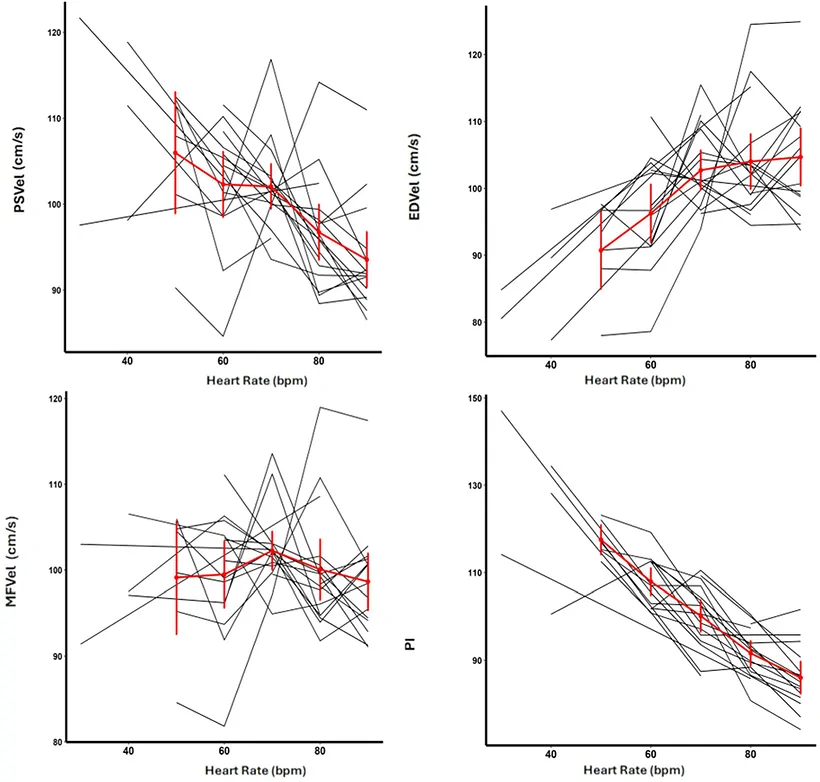
Standardised relationship between paced heart rate and haemodynamic parameters on TCD at follow‐up. Results are shown for each individual participant and the average across participants at each defined heart rate (red), as a percentage of the value at 80 bpm, for peak systolic velocity (a), end‐diastolic velocity (b), mean flow velocity (c), and pulsatiliy index (d).

**FIGURE A4:**
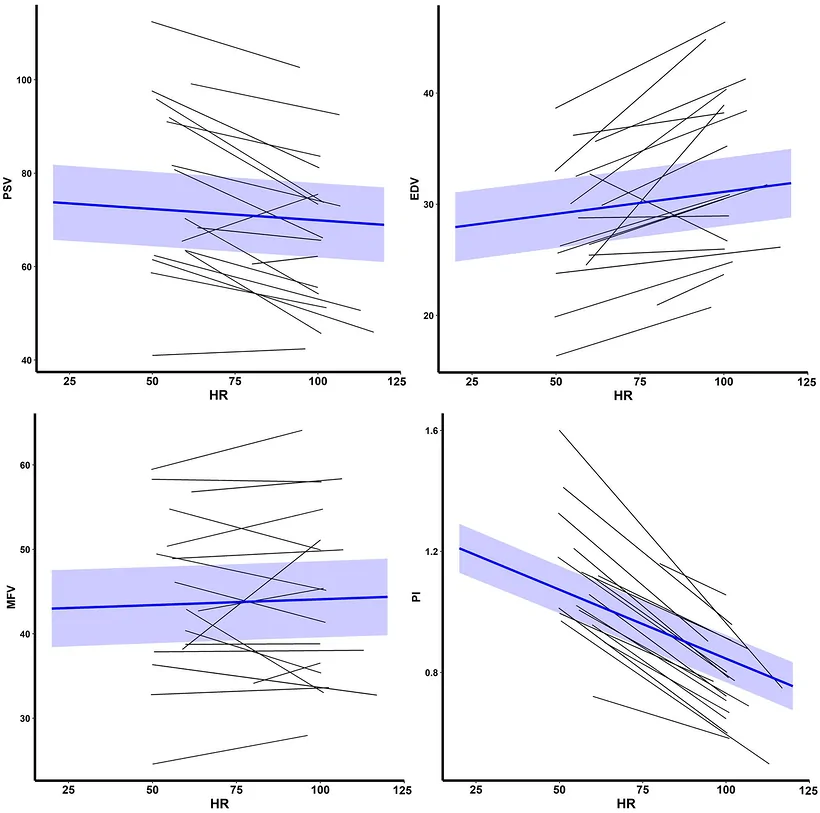
Standardised relationship between paced heart rate and blood pressure. Results are shown for each individual participant and the average across participants at each defined heart rate (red), as a percentage of the value at 80 bpm, for systolic blood pressure (a), diastolic blood pressure (b), mean blood pressure (c), and pulse pressure (d).

## Data Availability

The scientific data for the study may be shared upon appropriate requests to the chief investigator.

## 1

**TABLE A1:** Associations between heart rate cerebral blood flow velocity parameters.

Outcome	LME β	*P*	LME β adjusted	*P*
PSV	−0.48	<0.0001	−0.48	<0.0001
EDV	0.40	<0.0001	0.40	<0.0001
MFV	0.14	0.021	0.14	0.021
PI	−0.05	<0.0001	−0.05	<0.0001

Results are presented as the average β‐coefficient from linear mixed models. Results are derived from individual heart beats across all individuals, unadjusted and adjusted for age and sex, or age, sex and cardiovascular risk factors.

**TABLE A2:** Associations between demographic variables at baseline with the individual participant level associations (β‐coefficients) between heart rate and blood pressure parameters from linear regressions.

	HR–SBP	*P*	HR–DBP	*P*	HR–MBP	*P*	HR–PP	*P*
Age	0.190	0.754	0.232	0.495	0.218	0.555	−0.042	0.937
Male	−10.337	0.142	0.108	0.980	−3.374	0.451	−10.444	0.085
Diabetes	−17.362	0.156	1.727	0.812	−4.636	0.552	−19.089	0.067
Hypertension	−7.212	0.440	−0.539	0.920	−2.763	0.632	−6.673	0.415
Smoker	11.934	0.084	−3.264	0.429	1.802	0.690	15.198	0.005
Alcohol	−0.485	0.162	−0.112	0.584	−0.236	0.275	−0.373	0.226
PWV	−0.725	0.307	0.441	0.270	0.052	0.907	−1.166	0.042
Acute admission	−2.994	0.685	0.620	0.882	−0.584	0.897	−3.615	0.576
Systolic BP	0.063	0.773	0.117	0.299	0.099	0.491	−0.054	0.668
Diastolic BP	0.297	0.552	0.316	0.220	0.310	0.345	−0.019	0.949
PSV	0.374	0.211	0.295	0.063	0.321	0.094	0.080	0.705
EDV	0.797	0.132	0.622	0.023	0.680	0.041	0.175	0.645
MFV	0.653	0.140	0.512	0.025	0.559	0.045	0.141	0.655
PI	−27.469	0.274	−19.239	0.161	−21.982	0.180	−8.231	0.636

Reported values represent β‐coefficients from unadjusted linear regressions between predictors and the β‐coefficients from linear regressions listed in each column. BP, blood pressure; EDV, end diastolic velocity; MFV, mean flow velocity; PI, pulsatility index; PSV, peak systolic velocity; PWV, pulse wave velocity.

**TABLE A3:** Associations between demographic variables or mean cerebral haemodynamic variables at baseline with the individual participant level associations (β‐coefficients) between heart rate and cerebral blood velocity parameters from linear regressions, adjusted for risk factors and concurrent blood pressure.

	HR–PSV	*P*	HR–EDV	*P*	HR–MFV	*P*	HR–PI	*P*
Age	−0.114	0.529	0.059	0.380	0.001	0.988	−0.002	0.356
Male	−0.219	0.931	−0.500	0.592	−0.406	0.753	0.021	0.500
Diabetes	−0.363	0.922	−1.721	0.228	−1.268	0.511	0.002	0.970
Hypertension	−2.215	0.494	−1.353	0.266	−1.640	0.328	0.017	0.658
Smoker	0.170	0.978	1.626	0.476	1.141	0.716	−0.033	0.657
Alcohol	0.119	0.480	−0.052	0.401	0.005	0.951	0.003	0.160
PWV	−0.371	0.415	0.062	0.689	−0.082	0.691	−0.001	0.913
Acute admission	2.747	0.224	0.943	0.258	1.545	0.175	0.009	0.752

Reported values represent a β‐coefficient from the fully adjusted linear regression between each predictor and the β‐coefficients for each individual from an unadjusted linear regression for each individual participant as described in each column. EDV, end diastolic velocity; MFV, mean flow velocity; PSV, peak systolic velocity; PWV, pulse wave velocity.
